# A B‐Raf V600E gene signature for melanoma predicts prognosis and reveals sensitivity to targeted therapies

**DOI:** 10.1002/cam4.4491

**Published:** 2022-01-19

**Authors:** Kevin Yao, Emily Zhou, Chao Cheng

**Affiliations:** ^1^ Department of Electrical and Computer Engineering Texas A&M University College Station Texas USA; ^2^ Department of Biosciences Rice University Houston Texas USA; ^3^ Department of Medicine Baylor College of Medicine Houston Texas USA; ^4^ Dan L Duncan Comprehensive Cancer Center Baylor College of Medicine Houston Texas USA; ^5^ Institute for Clinical and Transcriptional Research Baylor College of Medicine Houston Texas USA

**Keywords:** B‐Raf, B‐Raf inhibitors, gene signature, Melanoma, prognostic prediction

## Abstract

**Background:**

B‐Raf V600E mutations account for about half of all skin cutaneous melanoma cases, and patients with this mutation are sensitive to BRAF inhibitors. However, aberrations in other genes in the MAPK/ERK pathway may cascade a similar effect as B‐Raf V600E mutations, rendering those patients sensitive to BRAF inhibitors. We rationalized that defining a signature based on B‐Raf pathway activity may be more informative for prognosis and drug sensitivity prediction than a binary indicator such as mutation status.

**Methods:**

In this study, we defined a B‐Raf signature score using RNA‐seq data from TCGA. A higher score is shown to not only predict B‐Raf mutation status, but also predict other aberrations that could similarly activate the MAPK/ERK pathway, such as B‐Raf amplification, RAS mutation, and EGFR amplification.

**Results:**

We showed that patients dichotomized by the median B‐Raf score is more significantly stratified than by other metrics of measuring B‐Raf aberration, such as mutation status, gene expression, and protein expression. We also demonstrated that high B‐Raf score predicts higher sensitivity to B‐Raf inhibitors SB590885 and PLX4720, as expected, but also correlated with sensitivity to drugs targeting other relevant oncogenic pathways.

**Conclusion:**

The BRAF signature may better help guide targeted therapy for melanoma, and such a framework can be applied to other cancers and mutations to provide more information than mutation status alone.

## Graphical abstract

Our BRAF score that captures BRAF V600E mutation pathway activity correlates with sensitivity to BRAF inhibitors, as well as other targeted therapies. The score is also able to predict prognosis and response to immunotherapy.

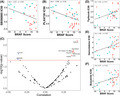

## 1 INTRODUCTION

Melanoma is the fifth most common cancer in both men and women in the United States.^1^ It is estimated that 101,280 adults will be diagnosed with melanoma this year.^1^ According to Surveillance, Epidemiology, and End Result (SEER) data, patients with primary localized melanoma tumors have a 99% 5‐year relative survival rate, however, this number decreases drastically to 27% for patients with metastatic melanoma tumors. Remarkably, 40%–60% of all melanoma patients exhibit B‐Raf mutations.^2^ In fact, just under half of metastatic melanoma tumors contain a B‐Raf mutation.^3^ B‐Raf (BRAF) is a serine/threonine protein kinase belonging to the RAF family and regulates the mitogen‐activated protein kinase/extracellular signal‐regulated kinase (MAPK/ERK) signaling pathway. The most prevalent BRAF mutation is the V600E mutation,^4^ especially in metastatic melanoma, where 70%–90% of patients express this specific point mutation.^3^ The BRAF V600E activating mutations promote the MAPK/ERK pathway resulting in uncontrolled cell proliferation.^5^

The FDA‐approved course of treatment for metastatic melanoma patients with BRAF mutations are BRAF inhibitors, which inhibits the BRAF kinase and blocks signaling to the MAPK/ERK pathway.^6^ Approved BRAF inhibitors for clinical use include vemurafenib (PLX4032), dabrafenib (GSK118438), encorafenib (LGX818), and sorafenib.^7^ There have been reports that BRAF V600E mutation status is not necessarily as good of a predictor to BRAF/MEK inhibitors response as previously thought.^8^, ^9^ Certain patients with BRAF mutations are insensitive to these inhibitors, attributed to increased cyclin D1 expression^10^ and MEK1 mutations,^11^ but other patients that are BRAF wild type have shown sensitivity to vemurafenib.^12^ These results are not surprising: the MAPK/ERK pathway consists of a multitude of signaling and activations from many different sources, so BRAF V600E mutation is not the only mechanism of BRAF activation. Such alternative pathways may be elucidated through study of the patient's transcriptome. In order for genomic aberrations to be oncogenic and affect cell functions, the aberration's effect must be reflected in the gene expression profile of the patient so that the oncogenic proteins may be transcribed. Aberrations that all affect the same pathway and cell function are expected to be captured through a consistent set of upregulated genes. As a result, there has been interest in developing a gene expression signature capturing the cumulative contributions of all possible pathways directly related to BRAF V600E mutation.

Gene signatures have been proposed to identify genes with differential regulation between BRAF V600E‐mutant and wild‐type patients in melanoma.^13^ Other gene signatures have been proposed to predict the prognosis of melanoma patients with and without BRAF V600E mutation.^14^, ^15^, ^16^ Still other gene signatures have been reported to predict the sensitivity, as well as eventual resistance, of melanoma patients to BRAF inhibitors.^17^, ^18^, ^19^ However, the gene sets defined in all these reports are largely dissimilar. Clinically, it is more useful to utilize a single set of genes in the signature, rather than multiple gene sets for different purposes, that is able to capture BRAF V600E mutation status, prognosis, and drug sensitivity. In addition, the gene signatures defined for prognosis and BRAF inhibitor prediction are often difficult to interpret in regard to BRAF activation, as they represent the contributions of many genes from multiple pathways. The development of a gene signature purely reflecting BRAF V600E activity has not yet been explored, but may provide biological insights on the impact of the pathway on prognosis and drug sensitivity. Lastly, the ability of gene signatures based on BRAF V600E in predicting sensitivities to drugs besides BRAF inhibitors has not been well defined. Such an analysis may pave the path for discovery of new drugs to target BRAF mutations and eliminate other drugs if they have no effect.

To address these current limitations, we defined a gene signature for the BRAF V600E mutation, rationalizing that a gene signature that characterizes the BRAF‐related pathways will be more informative of the sensitivity of melanoma tumors to anticancer drugs than BRAF mutations. We validated the efficacy of the signature in predicting BRAF V600E mutation, as well as other common genomic aberrations in the MAPK/ERK pathway. We then demonstrated the ability of the signature in predicting prognosis compared to BRAF mutation, expression, and protein levels. Lastly, we explored the correlation of the signature with various drugs and proposed mechanisms for drug sensitivity and resistance.

## 2 MATERIALS & METHODS

### 2.1 Datasets used in this study

The Cancer Genome Atlas (TCGA) RNA‐seq dataset for skin cutaneous melanoma (SKCM) was downloaded from Firehose (https://gdac.broadinstitute.org/). This dataset consisted of RSEM normalized gene expression data for 20,501 genes from 473 skin cutaneous melanoma samples. Of the SKCM samples, 103 are from primary tumors and 368 are from metastatic tumor samples. Besides gene expression, BRAF V600E mutation, copy number variation (CNV) data, and reverse phase protein assay (RPPA) data were used in this study. The mutations in 24,058 genes for each TCGA SKCM patient were downloaded as Mutation Annotation Format (MAF) files from Firehose. The TCGA CNV dataset was downloaded as a segmented copy number alteration (sCNA) file and contains CNV information for 23,311 genes, while the RPPA dataset was downloaded as raw protein expression data containing protein expression levels for 208 proteins and 355 samples. Both of these files were also downloaded through Firehose.

An additional four external datasets were used as validation. Of the four validation datasets, one was primary bulk tumor sample data, one was single cell tumor sample data, and two were cell line data. The bulk tumor sample data were downloaded from Gene Expression Omnibus (GEO) under accession ID GSE59455. This dataset contains 196 samples total and the mutation status information for BRAF V600E and NRAS mutations for 31 and 27 samples, respectively. Samples with incomplete data were removed from analysis. The single cell tumor sample data were downloaded from GEO under accession ID GSE81383. This dataset contained mutation status information of both BRAF V600E and NRAS for 307 and 141 samples, respectively. The cell line datasets were downloaded from the Genomics of Drug Sensitivity (GDSC) database (https://www.cancerrxgene.org) and the Cancer Cell Line Encyclopedia (CCLE) database (https://portals.broadinstitute.org/). Mutation status information was available for the 41 skin cancer cell lines in the GDSC dataset and 62 skin cancer cell lines in the CCLE dataset. Additional information for these datasets can be found in Table S1.

### 2.2 Defining the BRAF signature

We defined a weighted gene signature using primary SKCM RNA‐seq and BRAF V600E mutation status data from TCGA. Metastatic samples were left for the evaluation of its effectiveness. The BRAF mutation status was defined from the MAF files as follows: if the patient exhibited the V600E mutation in the BRAF gene, they are labeled as mutant (*n* = 27), and if the patient did not exhibit any mutations in the BRAF gene (including other possible mutations in the BRAF gene besides V600E), they are labeled as wild type (*n* = 26). To define the signature, we first log2 transformed the RSEM expression values of the genes to avoid extreme values. Each gene in the transformed gene expression profile is then used to predict the BRAF V600E mutation status (Y = 0 if BRAF wild type, 1 if BRAF V600E mutation) of the patient using a logistic regression model (Equation 1).

(1) Y=sigmoid(β0+β1∗gene+β2∗age+β3∗sex)

Note that the effect of clinical variables such as age and gender is also considered in the logistic model. Based on the model, we identified the top n genes that are most significantly upregulated (i.e., β_1_ > 0) to form the BRAF signature. The weights of signature genes were determined based on the *p*‐value for β_1_ using the following formula: **w_up_** = min((‐log10(*p*‐value), 10)/10. Note the log transformed *p*‐values were trimmed at 10 to avoid extreme values. We have evaluated the ability of gene signatures of different sizes (*n* = 20, 40, …. 800) for classifying BRAF‐mutant versus wild‐type melanoma samples based on cross‐validation. Our results indicated that the signature based on the top 200 most significant genes achieved the highest and most stable classification power, and was thus utilized through the rest of the study. These genes are listed in Table S2. Pathway enrichment analysis on this gene set was performed using the online tool DAVID, or the Database for Annotation, Visualization, and Integrated Discovery.

### 2.3 Calculation of BRAF score

Utilizing the top 200 most significant upregulated gene weights defined in the previous section, we calculated sample‐specific BRAF scores for patients in external gene expression datasets using a previously defined method named BASE (binding association with sorted expression).^20^ Initially, the external patient gene expression profile undergoes median normalization to mitigate the variation found in gene expression data. The median expression of each gene is subtracted from each tumor sample in the expression profile. Then, only the genes in the patient expression profile that are in the top 200 most significant upregulated gene weights are selected. Subsequently, this gene expression profile subset is sorted into decreasing order based on their median normalized gene expression, creating a ranked expression profile, **g** = {g_1_, g_2_, … g_200_}, with corresponding upregulated weights per gene, **w_up_** ={w_1_, w_2_, … w_200_}. Then, a foreground f(i) and background b(i) cumulative functions are applied to the data for both the upregulated and downregulated weights.

(2) $$f(i)=\frac{\sum_{k=1}^{i}g_{k}w_{k}}{\sum_{k=1}^{200}g_{k}w_{k}},1\leq i\leq 200$$

(3) $$b\left(i\right)=\;\frac{\sum_{k=1}^{i}g_{k}\left({1‐w}_{k}\right)}{\sum_{k=1}^{200}g_{k}\left({1‐w}_{k}\right)},1\leq i\leq 200$$

The foreground function captures the distribution influenced by highly informative genes, while the background function captures random distribution. The maximum difference between the two functions (denoted BRAF pre‐score) captures the biased distribution of BRAF signature genes in the sorted gene expression profiles for a tumor sample, similar to the enrichment score calculated in the gene set enrichment score (GSEA) analysis.^20^, ^21^

Next, the BRAF pre‐score is normalized by dividing by the null distribution BRAF pre‐score, determined based on 1000 permutations. In each permutation, the same process for calculating BRAF pre‐score was performed as described above, but the foreground and background functions are calculated using a random ordering of the patient gene expression profile. The normalized score, denoted BRAF score, summarizes the relative expression levels of BRAF signature genes and therefore indicates the BRAF pathway activity in the sample. Samples with high scores are expected to have a gene expression profile similar to patients with mutated BRAF V600E, capturing alterations in the MAPK/ERK pathway that is affected by the mutation. The result is a score that quantifies BRAF V600E mutation activity in the MAPK/ERK pathway.

### 2.4 Identification of gene amplification

To determine whether a sample exhibited amplification (AMP), we first halved and log2 transformed the raw TCGA CNV data downloaded from Firehose. Wild‐type copy number would then be transformed to log2(2/2) =0, and anything greater would indicate an amplification of copy number. We classified patients with CNV greater than log2(2.8/2) and Ch7 CNV segment length less than 151 Mb as amplification, and all other patients as wild type. Of note, 2.8 rather than 3 is used as the threshold for gain of additional copies to protect against tumor purity. Although some driver genes like MYCN in neuroblastoma and HER2 in breast cancer are amplified with large numbers of extra copies in the genome, most genes do not have such an extreme amplification. In the TCGA SKCM dataset, the few most amplified patients have a fold change of about 1.5, which is about 4 copy gains (Figure S1). With our threshold, we designate roughly 20% of the patients as having BRAF amplification. In addition, 151 Mb was used as the threshold for the length of the CNV segment so whole Ch7 gains are not considered as an amplification event. This process was used to determine the BRAF and EGFR amplification status for metastatic patients in the TCGA dataset. In total, 83 patients are determined to have BRAF amplification and 284 patients do not. In addition, 59 patients are determined to have EGFR amplification and 308 patients do not.

### 2.5 Association of genomic aberrations with BRAF score

We studied the relationship of various different BRAF mutations (such as V600E, V600K, etc.), BRAF amplification, NRAS mutation, and EGFR amplification with BRAF score in metastatic SKCM patients in the TCGA dataset. NRAS mutation status was determined from the TCGA MAF files, where 81 patients have at least one mutation in the NRAS gene (denoted as NRAS‐mutant) and 57 have no mutations in the NRAS gene (denoted as NRAS wild type). To determine the association of these aberrations with the BRAF score, we separated the samples into two groups: samples with the genomic aberrations and samples without the genomic aberrations. We then used a one‐sided Wilcoxon test to determine whether the group of patients with the genomic aberration had higher BRAF scores than the group of patients without the aberration. R package ggplot2 and pROC were used to generate the boxplots and ROC curves, respectively.

### 2.6 Survival analysis

Data for overall survival and time to event are contained in the TCGA dataset. We constructed univariate Cox regression models to determine the association of overall survival (OS) of the patient with the following factors: BRAF score, BRAF mutation status, BRAF expression levels, and BRAF protein expression, independently. The BRAF mutation status (“Mutant” or “Wild‐type”) could be directly used to divide patients into the BRAF‐mutant group and the BRAF wild‐type group. The BRAF score, BRAF expression levels, and BRAF protein expression are continuous variables, so we used the median of each of these variables as the threshold for dividing patients into two equal‐sized groups (high and low). The Kaplan–Meier method was used to plot the survival curves. The log‐rank tests were used to determine the difference between the two survival curves and statistical significance. All survival analyses were performed with the R library “survival,” and the samples were limited to the metastatic TCGA patients.

### 2.7 Drug sensitivity

Data for drug sensitivity were downloaded from GDSC database,^22^ which contained the sensitivity data of 41 melanoma cell lines with known BRAF V600E mutation status to 138 drugs. The sensitivity is represented by the concentration of drug needed to achieve half cancer inhibitory effect (IC50). A lower IC50 represents greater drug sensitivity. The dataset also contained gene expression data, which we used to calculate the BRAF score for each cell line. We used the Pearson method to quantify the correlation between our scores and the IC50 of drugs.

### 2.8 Tumor purity

Data used to calculate tumor purity for skin cutaneous melanoma in the TCGA dataset were downloaded from Firehose. Tumor purity was then calculated using the ESTIMATE algorithm.^23^

### 2.9 Immunotherapy data

Data for immunotherapy were downloaded from dbGaP. The accession code for this clinical study is phs000452.v3.p1. This dataset contains 42 patient samples each treated with checkpoint blockade immunotherapy.

## 3 RESULTS

### 3.1 Define gene signature to characterize oncogenic pathways downstream of BRAF

We first defined a gene signature based on the top 200 most significantly upregulated genes for BRAF V600E mutations in primary TCGA melanoma samples. These 200 genes are listed in Table S2. Then, we analyzed the pathway enrichment of this gene set using functional annotation clustering of the gene set's Gene Ontology (GO) (Table S3) and Kyoto Encyclopedia of Genes and Genomes (KEGG) pathway enrichment (Table S4). GO analysis revealed that this gene set contained genes highly enriched for the positive regulation of MAPK activity and the associated pathway results, such as proliferation, as expected. Interestingly, genes related to Na+ and K+ ion transportation are also enriched in this gene set, this result may be understood since the MAPK/ERK cascade has been reported to control ion transporter activity for kidney cells.^24^ ^(p1)^ Additionally, KEGG pathway enrichment analysis revealed that the gene set contained genes highly enriched in melanoma and RAS signaling pathway. Furthermore, we observed that genes in the cAMP signaling pathway were enriched in our gene set, which could be explained by the well‐established cross talk between the MAPK/ERK pathway and cAMP pathway.^25^

**FIGURE 1:**
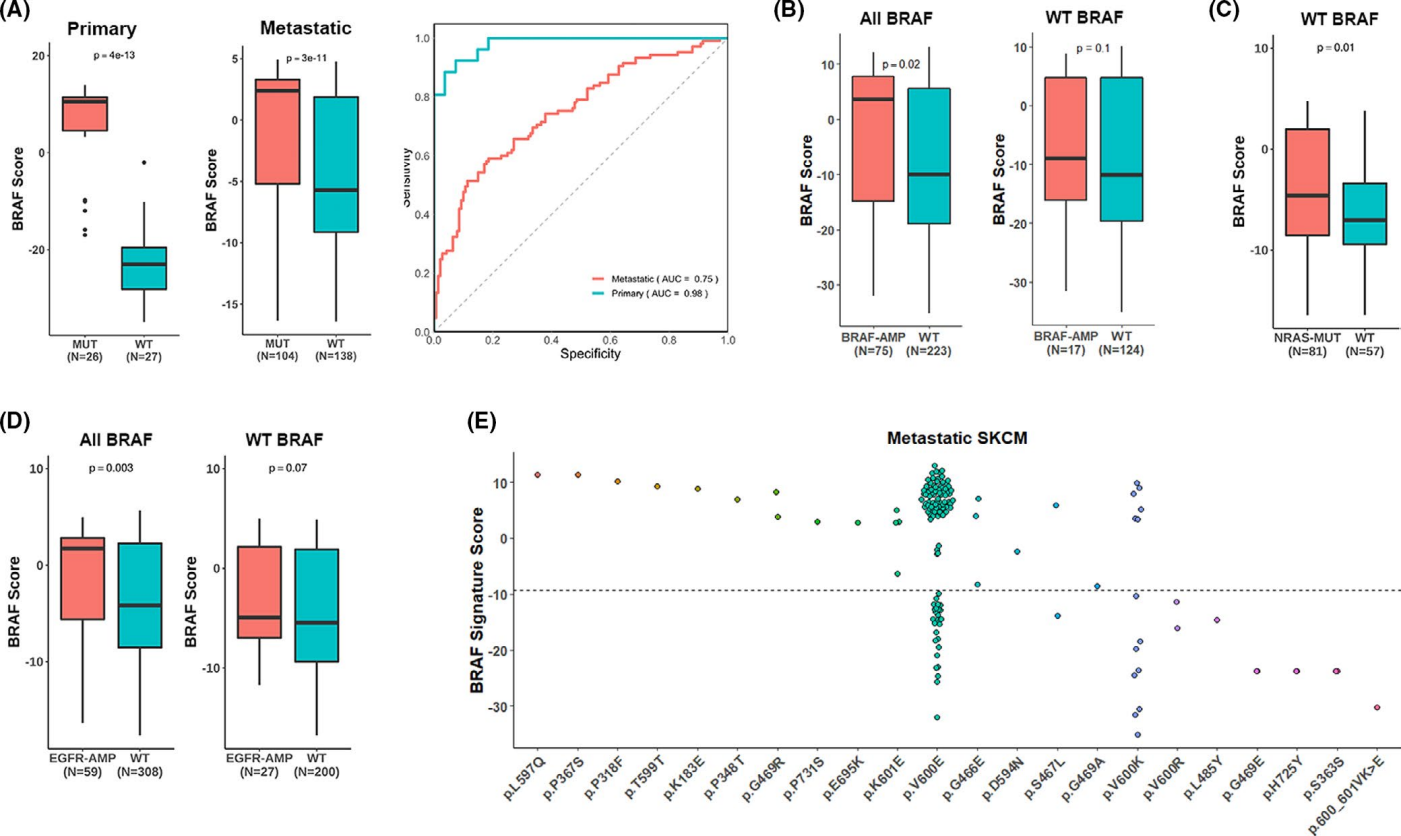
BRAF score recapitulates BRAF‐related pathway activity. (A) Patients with mutated BRAF V600E have significantly higher BRAF score compared to patients with wild‐type BRAF, in both primary and metastatic patients in the TCGA dataset. (B) Patients with BRAF amplification have higher BRAF scores than those without the amplification. This relationship holds for all BRAF patients and wild‐type BRAF patients. (C) NRAS mutations are associated with higher BRAF scores compared to wild‐type NRAS for wild‐type BRAF patients. (D) Patients with EGFR amplification (AMP) are associated with higher BRAF scores compared to patients without amplified EGFR. (E) BRAF score can quantify the functional impact of various other BRAF missense, silent, and splice site mutations besides the V600E mutation

Having shown that our gene signature captures activity in the MAPK/ERK pathway, we utilized the gene signature to calculate the BRAF score for each patient in our datasets. To demonstrate the ability of this BRAF score to encapsulate pathway activity, we showed that BRAF score can identify BRAF V600E mutation status and additional gene aberrations within TCGA melanoma tumor samples. In order to assess the association between BRAF score and mutation status, we compared BRAF scores between BRAF‐mutant and wild‐type (WT) samples. As shown in Figure 1A, BRAF‐mutant samples correctly exhibited significantly higher BRAF scores compared to wild‐type samples in both primary (*p* = 4e‐13) and metastatic (*p* = 3e‐11) samples, indicating increased pathway expression in patients with the BRAF V600E mutation. Interestingly, BRAF scores in metastatic tumor samples appear to be overall lower than BRAF scores in primary tumor samples even though there is higher BRAF expression in metastatic mutant samples. A potential mechanism for this difference in scores is that metastatic samples tend to have lower tumor purity compared to primary samples (Figure S2). The difference in tumor purity is to be expected due to the more complicated anatomic cell composition of metastatic tissue compared to primary tumors. The non‐cancer cells may tend to bias the score to be lower. Another possible explanation is since we defined the gene signature in the primary samples, the score is fitted to distinguish BRAF‐mutant and WT samples in that dataset with the greatest magnitude. However, in other datasets, the magnitudes of the scores are expected to be lower since the gene weights were not fitted to provide the greatest discrimination power for that dataset. Finally, the ability of the BRAF score to differentiate between BRAF‐mutant samples and wild‐type samples is further demonstrated through the receiving operator characteristic (ROC) curve (primary AUC = 0.98, metastatic AUC = 0.75), showing that BRAF score is predictive of BRAF V600E mutation status.

Beyond BRAF V600E mutation, we explored the association of BRAF score with additional genomic aberrations, specifically BRAF amplification (Figure 1B), NRAS mutation (*p* = 0.01, Figure 1C), and EGFR amplification (*p* = 0.003, Figure 1D). BRAF scores are higher for BRAF amplification as compared to BRAF non‐amplification (*p* = 0.02, Figure 1B). To control for the possibility that BRAF AMP status is dependent on BRAF mutation status and avoid artificially skewing the data, we demonstrated that the same correlation between BRAF amplification and non‐amplification exists in BRAF wild‐type samples (*p* = 0.1). We also showed that NRAS mutants generally produced higher BRAF scores than NRAS wild type in BRAF wild‐type samples (*p* = 0.01, Figure 1C). Usually, NRAS mutations and BRAF mutations are mutually exclusive,^26^ so BRAF‐mutant patients with NRAS mutations were not studied. Samples with EGFR amplification are also associated with higher BRAF scores compared to EGFR wild‐type samples (*p* = 0.003, Figure 1D). The relationship also holds for BRAF wild‐type patients (*p* = 0.07). Since these genomic aberrations activate the MAPK/ERK pathway, it is expected that they exhibit higher BRAF scores.

Finally, as shown in Figure 1E, BRAF score can quantify the functional impact or lack of impact of other mutations besides BRAF V600E such as in‐frame deletions and other point mutations on BRAF activity. The results in this figure parallel the findings in previous papers.^27^, ^28^, ^29^ For example, the sample with the p.L597Q exhibits the highest BRAF score in comparison to wild‐type BRAF scores (Figure 1E). This parallels the finding by Smiech et al. that states that the p.L597Q mutation is found in the activation segment.^27^ The presence of many other BRAF hotspot alterations that exhibited higher BRAF scores than V600E mutation prompted us to re‐define the BRAF gene signature by comparing between samples with V600E and other hotspot BRAF mutations reported in COSMIC (recurrence of >= 10 patients) with wild‐type samples. After recalculating the BRAF score we found that the results were consistent with the signature based only on V600E mutations (Figure S3). This result is due to the vast majority of BRAF mutations being V600E mutations. Of note are a considerable fraction of patients with the V600E mutation that have scores less than –10. To explore why, we noticed that the promoter methylation levels were significantly higher in patients with the V600E mutation but lower BRAF scores compared to those with higher BRAF scores (Figure S4). These patients with higher promoter methylation levels would thus have lower expression of BRAF and lower BRAF pathway activity. This epigenetic silencing provides a possible explanation why some patients have such low scores despite having the V600E mutation.

### 3.2 BRAF score is associated with patient prognosis

**FIGURE 2:**
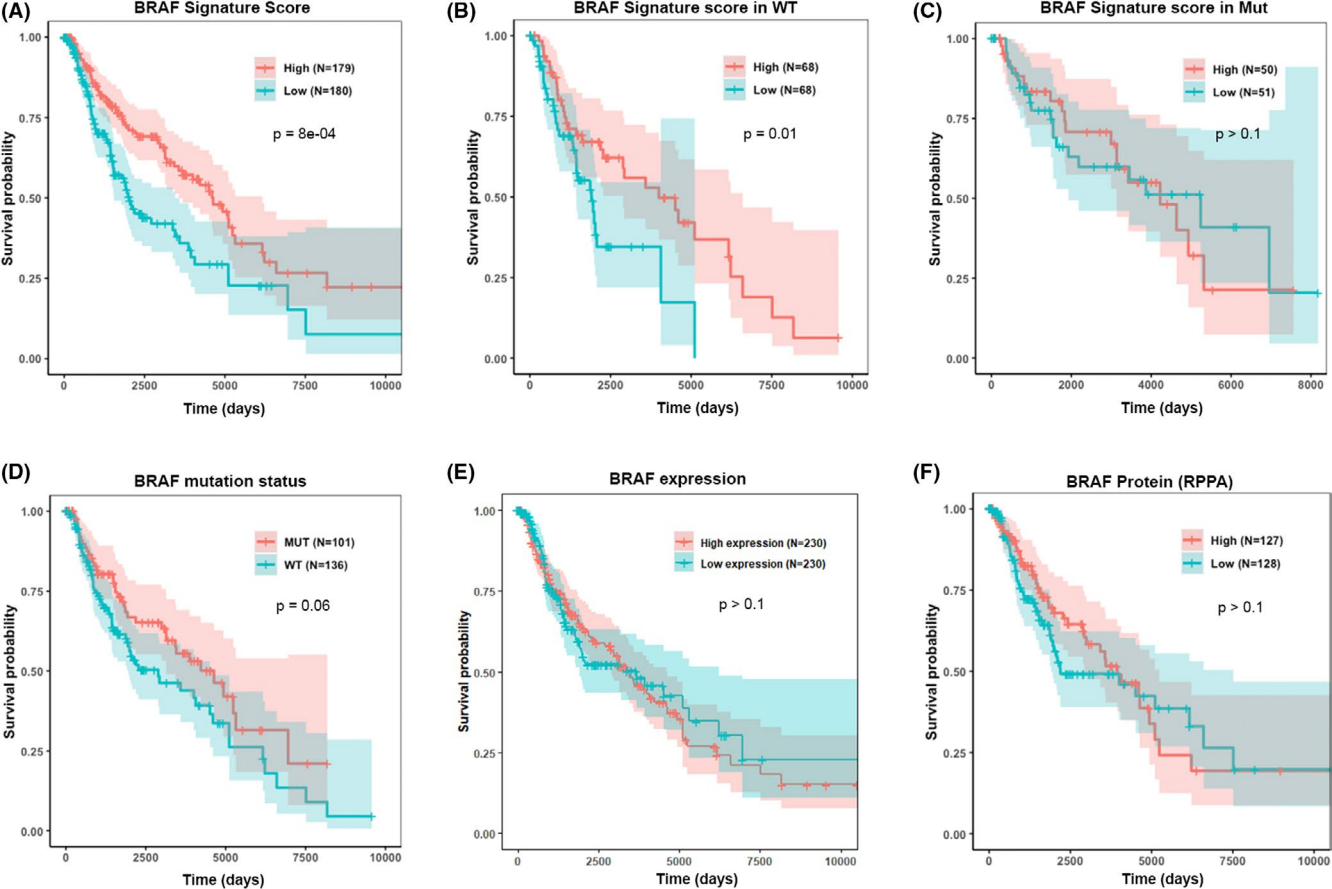
Association between BRAF score and patient prognosis. (A) Median BRAF score significantly dichotomizes patients into separate risk groups. (B,C) Median BRAF score also dichotomizes patients without the BRAF V600E mutation (B), but cannot dichotomize patients with the mutation (C). (D–F) BRAF mutation status, median BRAF expression, and median protein expression, respectively, are unable to dichotomize patients into statistically significant risk groups, showing the advantage of our BRAF score

Having shown the ability of the BRAF score to recapitulate pathway activity, we next demonstrated the prognostic value of the BRAF score. TCGA melanoma samples were dichotomized into high and low BRAF score groups using the median BRAF score as the threshold. Utilizing the dichotomized scores, we constructed a univariate Cox regression model and generated survival curves. Patients with high scores exhibited significantly better prognosis compared to patients with low scores (*p* = 8e‐04, Figure 2A). Similar results were observed in BRAF wild‐type only samples (*p* = 0.01, Figure 2B), but no significant correlation was found in BRAF‐mutant only samples (*p* > 0.1, Figure 2C). This result can be understood since the BRAF V600E mutation continuously activates the downstream MAPK/ERK pathway, so the activity of other BRAF activators captured by the BRAF score (such as EGFR amplification or RAS mutation) is not as important.

To demonstrate the advantage of our score, we also explored the prognostic value of BRAF V600E mutation status and found that mutation status was not as significant for predicting prognosis (*p* = 0.06, Figure 2D). We note that patients with BRAF MUT were also observed to have better survival than patients with wild‐type BRAF, agreeing with our result that patients with higher BRAF score have better survival. Furthermore, BRAF mRNA expression levels and BRAF protein levels were examined to determine their association with prognosis. However, the results were not significant (*p* > 0.1, Figure 2E,F). BRAF score appeared to predict survival better than mutation status, BRAF gene expression levels, and BRAF protein levels.

### 3.3 BRAF score reflects the BRAF mutation status in melanoma and cancer cell lines

**FIGURE 3:**
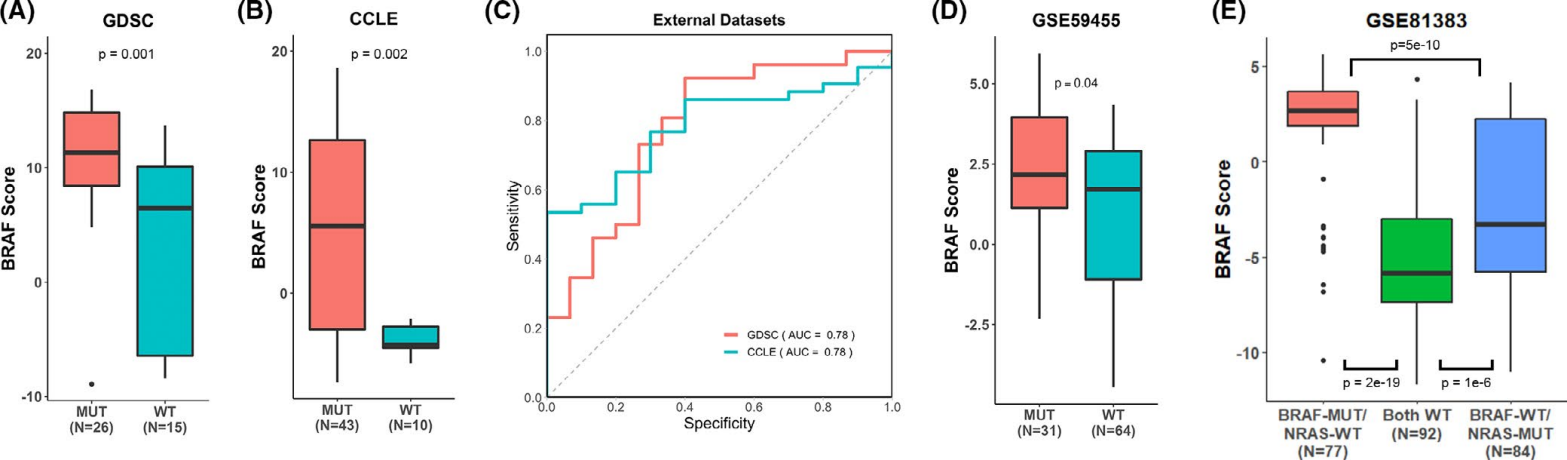
BRAF score predicts BRAF mutation status in external datasets. (A) BRAF‐mutant samples exhibit significantly higher BRAF scores than wild‐type (wild type) samples in the GDSC dataset (Wilcoxon). (B) BRAF‐mutant samples exhibit significantly higher BRAF scores compared to wild‐type samples in CCLE dataset (Wilcoxon). (C) BRAF score differentiates between BRAF mutants and wild‐type samples with relative accuracy. (D) BRAF‐mutant samples have significantly higher BRAF scores in the GSE59455 dataset. (E) BRAF‐mutant samples show significantly higher BRAF scores compared to wild‐type and NRAS‐mutant samples in single cell data (Wilcoxon). The number of samples for each group is located in the label

We further demonstrated BRAF score's ability to differentiate between BRAF V600E mutants and wild‐type samples by comparing the BRAF scores between the two groups in two cell line datasets (GDSC and CCLE) and two external tumor datasets (GSE59455 and GSE81383). Similar to the previous results, higher BRAF scores are significantly associated with BRAF‐mutant samples in GDSC (*p* = 0.001) and CCLE (*p* = 0.002) samples (Figure 3A,B). Furthermore, BRAF score was able to accurately predict BRAF mutation status in GDSC (AUC = 0.78) and CCLE (AUC = 0.78) samples (Figure 3C). We further confirmed BRAF scores’ ability to differentiate between BRAF‐mutant and wild‐type samples using the GSE59455 dataset. To do so, we separated the tumor samples into BRAF mutant, which only contain BRAF V600E mutations, and wild type. It is noted that within this dataset there also contains samples with only NRAS mutations, however, we did not include those samples into either category. As shown in Figure 3D, patients with BRAF V600E mutations exhibited significantly higher BRAF scores compared to patients who were wild type for BRAF mutations (*p* = 0.04). To determine the capability of BRAF score to differentiate between BRAF mutant, NRAS mutant, and wild‐type samples, we segregated samples in the GSE81383 dataset into samples with BRAF mutation and wild‐type NRAS, samples with NRAS mutation and wild‐type BRAF, and samples with both wild‐type BRAF and NRAS (Figure 3E). BRAF score is significantly higher in patients with BRAF mutations (but NRAS wild type) than patients who were wild type for both BRAF and NRAS (*p* = 2e‐19), as expected. Furthermore, samples that contain NRAS mutations (but BRAF wild type) have lower BRAF scores than BRAF mutants (*p* = 5e‐10) but significantly higher BRAF scores than wild‐type only samples (*p* = 1e‐6). This observation demonstrates that both BRAF V600E mutations and NRAS mutations activate downstream pathways, but the extent in which each mutation influences activity differs. Such a result may be explained by the different effects of mutations in different NRAS loci. In fact, Murphy et al. have reported that NRAS Q61R, Q61K, and Q61L mutations cause melanoma in mice but not G12D, G13D, G13R, Q61H, or Q61P.^30^ Another study also supported those results, reporting that mutations in codon 61 correlated with poor prognosis while mutations in codon 12 and 13 did not.^31^ Thus, some samples with NRAS mutations may not be at the locus that activates BRAF, causing lower score.

### 3.4 BRAF score is predictive of cell line sensitivity to targeted drugs

**FIGURE 4:**
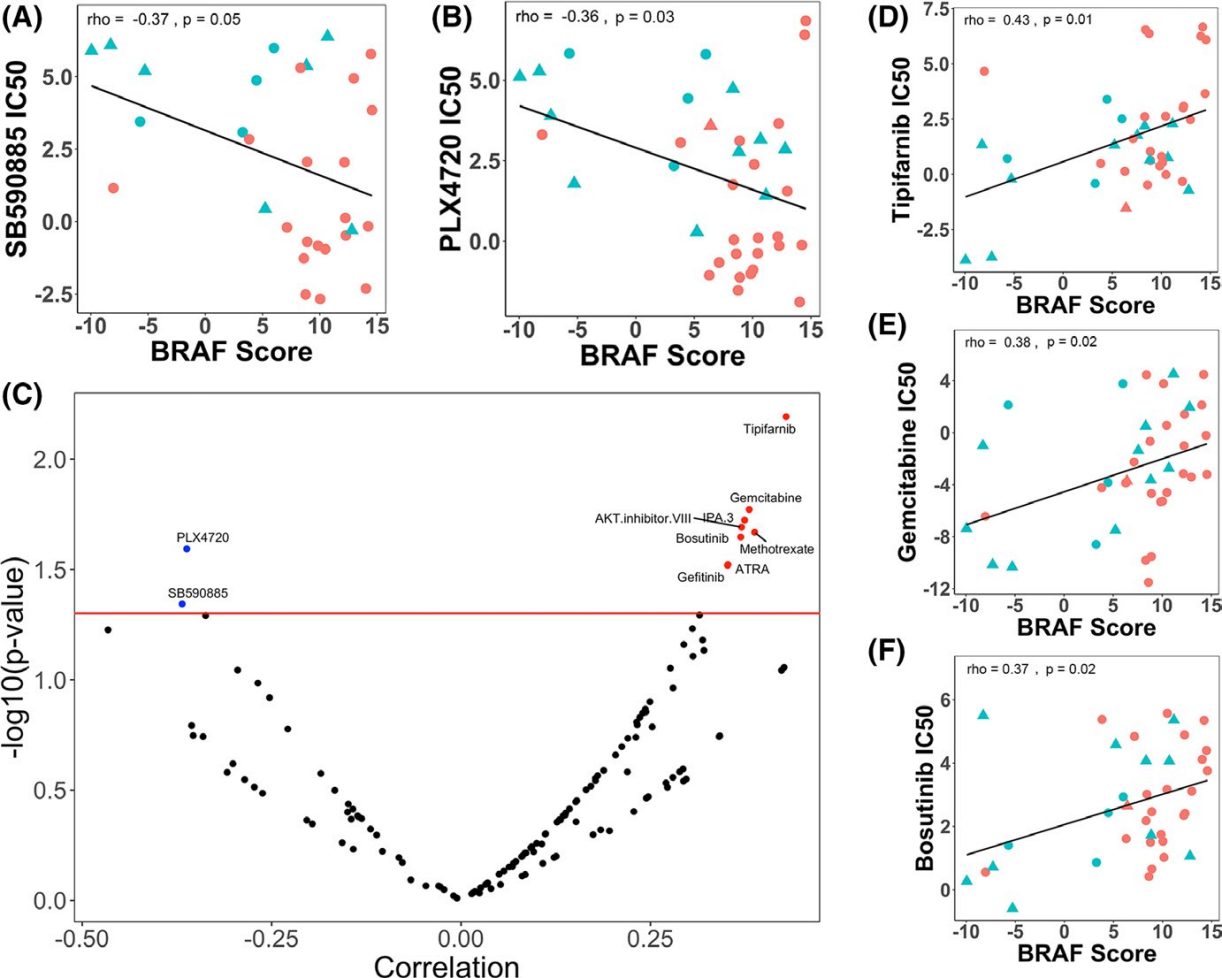
Correlation between BRAF score and drug sensitivity. (A) BRAF score exhibits a significant negative correlation with the IC50 of SB590885. Blue samples are BRAF wild type and red samples are BRAF V600E mutants. Triangle‐shaped samples exhibit RAS mutation. (B) BRAF score exhibits a significant negative correlation with the IC50 of PLX4720. (C) Volcano plot of all the rho values and *p*‐values of every drug in GDSC. Dots colored black are not significant. Correlation value is calculated using the Pearson method. (D–F) BRAF score exhibits a significant positive correlation with the IC50 of tipifarnib, gemcitabine, and bosutinib

Given the availability of BRAF inhibitors and other drugs used for targeted therapy, we explored the relationship of BRAF score with various inhibitors targeting the MAPK pathway. To determine the possible mechanisms for drug sensitivity, GDSC melanoma samples were segregated based on their BRAF and RAS (KRAS, NRAS, and HRAS) mutation status. The BRAF scores of these samples were then correlated with the IC50 of the BRAF inhibitors available in the GDSC database. As expected, there was a negative correlation between BRAF score and the IC50 of BRAF inhibitors SB590885 (rho = −0.37, *p* = 0.05) and PLX4720 (rho = −0.36, *p* = 0.03) in Figure 4A,B. This negative correlation revealed higher sensitivity of cell line samples with higher BRAF scores to the BRAF inhibitors.

To further explore the sensitivity of other anticancer drugs, we examined the association of BRAF score with the IC50 of all 138 drugs in the GDSC dataset. The *p*‐value and correlation of every drug from the GDSC dataset with BRAF score are shown in the volcano plot in Figure 4C. Due to their high and significant correlation values with BRAF score, we decided to more closely examine tipifarnib, gemcitabine, and bosutinib. BRAF score is positively correlated with the IC50 of tipifarnib (*p* = 0.01), gemcitabine (*p* = 0.02), and bosutinib (*p* = 0.02), indicating cell line resistance to the drugs (Figure 4D–G).

### 3.5 BRAF score is associated with patient response to CTLA4 immunotherapy

**FIGURE 5:**
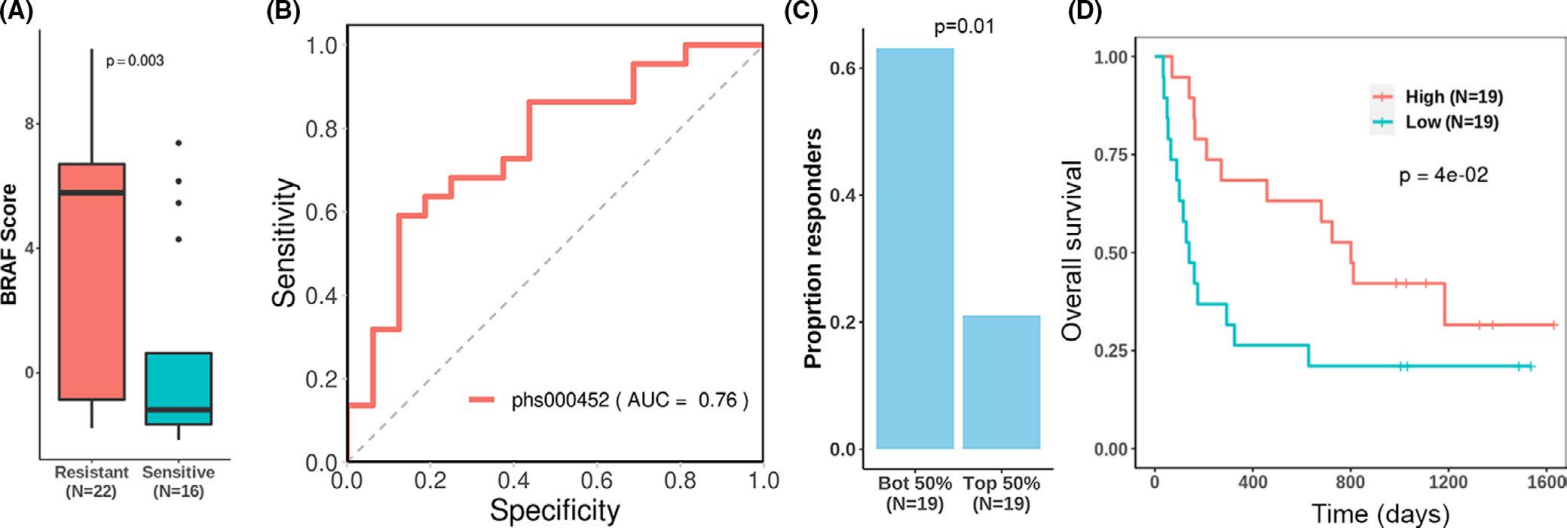
Association of BRAF score with immunotherapy. (A) Immunotherapy‐resistant samples exhibit significantly higher BRAF score (*p*‐value determined via Wilcoxon). (B) BRAF score differentiates between immunotherapy‐resistant and sensitive patients with fairly high accuracy. (C) About 60% of patients with a BRAF score lower than the median responded to immunotherapy, whereas only about 20% of patients with a BRAF score higher than the median responded to immunotherapy. The *p*‐value is determined via the Fisher's exact test. (D) Median BRAF expression significantly dichotomizes the survival of patients treated with immunotherapy

Using available immunotherapy clinical study data, we explored the association of BRAF score with patient response to CTLA4 immunotherapy by comparing the BRAF scores between immunotherapy‐resistant and immunotherapy‐sensitive patients in the phs000452 dataset. As shown in Figure 5A, BRAF scores of CTLA4 immunotherapy‐resistant samples are significantly higher than BRAF scores of immunotherapy‐sensitive patient samples (*p* = 0.003). To further examine BRAF scores ability to differentiate between CTLA4‐sensitive and insensitive patients, we constructed a ROC curve to demonstrate that BRAF score can predict patient response (AUC = 0.76, Figure 5B). Multivariate logistic regression indicated that BRAF score remains significant in predicting response after considering major clinical factors, including age, gender, presence of other therapies like BRAF inhibitors. (Table S5).

In clinical settings, physicians will likely make decisions based on whether a patient is classified with a high or low BRAF score, so we further explored the ability of these classifications in reflecting CTLA4 immunotherapy sensitivity and patient prognosis. Patients in the phs000452 dataset are dichotomized into high and low BRAF scores based on their median, and we found that over 60% of patients with a low BRAF score were sensitive to CTLA4 immunotherapy, compared to about 20% of those with a high BRAF score were sensitive (Figure 5C). Furthermore, the clinical trial patients were separated into groups with high BRAF score and low BRAF score using the median BRAF score as the threshold. As shown in Figure 5D, patients with low BRAF scores have significantly worse prognosis. This is consistent with previous findings in this paper.

## 4 DISCUSSION

In this study, we defined a gene signature based on the top 200 most significantly upregulated genes in BRAF V600E versus BRAF wild‐type samples from primary skin cutaneous melanoma data in the TCGA dataset. The ability of the signature to capture activity in the BRAF pathway is validated by demonstrating the differential distribution in BRAF score for other genomic aberrations in the MAPK/MEK pathway versus their wild‐type counterparts. In addition, the prognostic value of the BRAF score was shown: compared to other BRAF markers and indicators, such as BRAF mutation, expression, and protein expression, our BRAF score is the most significant in predicting the prognosis of patients. Lastly, we explored the relationship between BRAF score and the sensitivity of patients to various drugs, including BRAF inhibitors and other common anticancer compounds.

In our first step, we aimed to demonstrate that the BRAF score quantified the activity of the BRAF V600E mutation in the MAPK/ERK pathway. We showed that BRAF amplification is correlated with higher BRAF scores, even for BRAF wild‐type patients. This is reasonable since gene amplification is known to correlate with an overproduction of protein, thus causing an increased activation of BRAF that may cascade similar effects as the activating BRAF V600E mutation. We also observed that mutations in the NRAS protein resulted in higher BRAF scores. NRAS is a GTPase responsible for activating members of the RAF family (including BRAF) by binding GDP to GTP, but NRAS normally remains GDP bound and inactive. However, mutations in NRAS causes loss of enzymatic function, resulting in GTP being constitutively bound to NRAS and perpetually activating RAF.^32^ Thus, if our BRAF score was to reflect BRAF pathway activity, we should expect higher scores for NRAS‐mutant patients, even if the patient was BRAF wild type, which is supported by Figure 1C. In addition, patients with epidermal growth factor receptor (EGFR) amplification were observed to have higher BRAF scores than those without the amplification. EGFR is a receptor tyrosine kinase (RTK) that binds EGF and growth factor‐alpha (as well as other ligands) to autophosphorylate tyrosine residues in the receptor, which eventually signals GDP to GTP binding in RAS and activation of BRAF.^33^ Thus, amplification of this receptor should be positively correlated with increased BRAF activity, which is observed in Figure 1D.

We also explored the correlation of the BRAF score with various drugs in the GDSC dataset. The observation that higher BRAF score correlates with better sensitivity to SB590885 and PLX4720 is expected: higher BRAF score is correlated with the presence of the BRAF V600E mutation, and BRAF inhibitors are known to be effective in patients with the BRAF V600E mutation.^34^, ^35^ It is important to note that although the majority of the samples with high BRAF scores are BRAF V600E mutant, there are a few BRAF wild‐type samples with high BRAF scores. Whereas BRAF mutation status would not have been informative of these samples’ sensitivity to the drugs, the BRAF score was able to predict the lower than expected IC50 of these wild‐type samples. Even though a patient may not have BRAF mutation, inhibition of BRAF may still be effective in initially controlling the cancer if the BRAF pathway was activated via alternative pathways, such as BRAF amplification, NRAS mutation, etc. These wild‐type samples contain RAS mutations, a possible explanation for the high BRAF scores and increased sensitivity to the BRAF inhibitors, since the RAS isoforms functions to signal activation of BRAF.^32^ Such other pathways may explain the observation that PLX4720 is able to somewhat inhibit the BRAF kinase activity for certain cell lines without the BRAF V600E mutation.^36^

We further found that higher BRAF score predicted worser sensitivity for tipifarnib, gemcitabine, and bosutinib, which are understood as follows. First, tipifarnib is an RAS inhibitor.^37^ It has been reported that BRAF activation engages in a negative feedback loop with RAS.^38^ Thus, if a patient has a higher BRAF activation, RAS is more inhibited, so inhibiting RAS with tipifarnib would not affect the pathway any further. BRAF will continue to be activated and the cell will continue overexpressing the pathway that leads to tumor cell proliferation, survival, and metastasis. Second, gemcitabine is an inhibitor of DNA synthesis.^39^ Our result supports a previous report in which gemcitabine has been shown to have lower sensitivity in patients with higher MAPK/ERK pathway activation.^40^ Lastly, bosutinib is a Src kinase inhibitor.^41^ Src and RTKs, such as EGFR, have been shown to activate each other.^42^ At the same time, EGFR is negatively controlled by BRAF activity.^38^ Thus, patients with higher BRAF scores should have inhibited EGFR activity, which then inhibits Src. Again, if bosutinib inhibits a part of the pathway that is not activated, there will be a limited effect on the cancer cell, as its tumorigenesis depends on an alternate pathway that is not addressed by the drug.

Lastly, the BRAF score's clinical utility and applicability to patients in clinical trials were demonstrated in the phs000452 dataset. A higher BRAF score was found to be correlated with a worser response rate to CTLA4 immunotherapy. BRAF mutations are known to promote cancer evasiveness and enhance oncogenic activity, providing a possible mechanism of resistance to PD‐1/PD‐L1 immunotherapy.^43^ Similarly, patients with greater BRAF activity (higher BRAF score) would be more likely to be resistant to other types of immunotherapy like anti‐CTLA4, which supports our results. We note that in clinical situations, our BRAF score may save clinicians’ time in sequencing the gene mutation profile of the patient. Instead, the BRAF score can be calculated with just the gene expression data. The BRAF score may warn physicians to prescribe more aggressive forms of treatment especially if the patient has a high score and is unlikely to respond.

## 5 CONCLUSION

In summary, we developed a gene signature based on BRAF V600E mutation that captures pathway activity, predicts prognosis, and correlates with sensitivity to BRAF inhibitors and other targeted therapies. Although we were able to study drug response in cell lines, this study was limited by the unavailability of open‐source BRAF inhibitor response data in clinical trials with patients. Once such data becomes available, the ability of BRAF score to predict patient response can be studied. The methodology described in this study may help guide clinical decisions in prescribing BRAF inhibitors and can be generalizable and applied to other cancers and targeted therapies.

## AUTHOR CONTRIBUTIONS

CC is responsible for the study concept and design. CC, KY, and EZ are responsible for the acquisition of data. KY and EZ are responsible for the analysis and interpretation of data. KY and EZ are responsible for the drafting and revising of the manuscript. CC is responsible for the study supervision. All authors have approved the final version of the manuscript.

## CONFLICT OF INTEREST

The authors have declared that no conflict of interest exists.

## Funding statement

This work was supported by the Cancer Prevention Research Institute of Texas (CPRIT) (RR180061 to CC) and the National Cancer Institute of the National Institutes of Health (1R21CA227996 to CC). CC is a CPRIT Scholar in Cancer Research.

## Supporting information

Fig S1‐S4

Table S1

Table S2

Table S3

Table S4

Table S5

## Data Availability

All data generated or analyzed during this study are included in this published article and its supplementary information files.

## References

[1] Siegel RL , Miller KD , Fuchs HE , Jemal A . Cancer statistics, 2021. CA Cancer J Clin. 2021;71(1):7‐33. doi:10.3322/caac.21654 33433946

[2] Hannan EJ , O’Leary DP , MacNally SP , et al. The significance of BRAF V600E mutation status discordance between primary cutaneous melanoma and brain metastases: the implications for BRAF inhibitor therapy. Medicine (Baltimore). 2017;96(48):e8404. doi:10.1097/MD.0000000000008404 29310328 PMC5728729

[3] Kong BY , Carlino MS , Menzies AM . Biology and treatment of BRAF mutant metastatic melanoma. Melanoma Manag. 2016;3(1):33‐45. doi:10.2217/mmt.15.38 30190871 PMC6097549

[4] Cheng L , Lopez‐Beltran A , Massari F , MacLennan GT , Montironi R . Molecular testing for BRAF mutations to inform melanoma treatment decisions: a move toward precision medicine. Modern Pathol. 2018;31(1):24‐38. doi:10.1038/modpathol.2017.104 PMC5758899 29148538

[5] Ascierto PA , Kirkwood JM , Grob JJ , et al. The role of BRAF V600 mutation in melanoma. J Transl Med. 2012;10:85. doi:10.1186/1479-5876-10-85 22554099 PMC3391993

[6] Morris V , Kopetz S . BRAF inhibitors in clinical oncology. F1000prime Rep. 2013;5:11. doi:10.12703/P5-11 23585929 PMC3619157

[7] Sanchez JN , Wang T , Cohen MS . BRAF and MEK Inhibitors: Use and Resistance in BRAF‐Mutated Cancers. Drugs. 2018;78(5):549‐566. doi:10.1007/s40265-018-0884-8 29488071 PMC6080616

[8] Rinehart J , Adjei AA , LoRusso PM , et al. Multicenter phase II study of the oral MEK inhibitor, CI‐1040, in patients with advanced non‐small‐cell lung, breast, colon, and pancreatic cancer. J Clin Oncol. 2004;22(22):4456‐4462. doi:10.1200/JCO.2004.01.185 15483017

[9] LoRusso PM , Adjei AA , Varterasian M , et al. Phase I and pharmacodynamic study of the oral MEK inhibitor CI‐1040 in patients with advanced malignancies. J Clin Oncol. 2005;23(23):5281‐5293. doi:10.1200/JCO.2005.14.415 16009947

[10] Smalley KSM , Lioni M , Palma MD , et al. Increased cyclin D1 expression can mediate BRAF inhibitor resistance in BRAF V600E‐mutated melanomas. Mol Cancer Ther. 2008;7(9):2876‐2883. doi:10.1158/1535-7163.MCT-08-0431 18790768 PMC2651569

[11] Carlino MS , Fung C , Shahheydari H , et al. Preexisting MEK1 ^P124^ mutations diminish response to BRAF inhibitors in metastatic melanoma patients. Clin Cancer Res. 2015;21(1):98‐105. doi:10.1158/1078-0432.CCR-14-0759 25370473

[12] Krayem M , Journe F , Wiedig M , et al. Prominent role of cyclic adenosine monophosphate signalling pathway in the sensitivity of WTBRAF/WTNRAS melanoma cells to vemurafenib. Eur J Cancer. 2014;50(7):1310‐1320. doi:10.1016/j.ejca.2014.01.021 24559688

[13] Kannengiesser C , Spatz A , Michiels S , et al. Gene expression signature associated with *BRAF* mutations in human primary cutaneous melanomas. Mol Oncol. 2008;1(4):425‐430. doi:10.1016/j.molonc.2008.01.002 19383316 PMC5543835

[14] Hauschild A , Larkin J , Ribas A , et al. Modeled prognostic subgroups for survival and treatment outcomes in *BRAF* V600–mutated metastatic melanoma: pooled analysis of 4 randomized clinical trials. JAMA Oncol. 2018;4(10):1382. doi:10.1001/jamaoncol.2018.2668 30073321 PMC6233771

[15] Song LB , Zhang QJ , Hou XY , et al. A twelve‐gene signature for survival prediction in malignant melanoma patients. Ann Transl Med. 2020;8(6):312. doi:10.21037/atm.2020.02.132 32355756 PMC7186619

[16] Mann GJ , Pupo GM , Campain AE , et al. BRAF mutation, NRAS mutation, and the absence of an immune‐related expressed gene profile predict poor outcome in patients with stage III melanoma. J Invest Dermatol. 2013;133(2):509‐517. doi:10.1038/jid.2012.283 22931913

[17] Koroknai V , Patel V , Szász I , Ádány R , Balazs M . Gene expression signature of BRAF inhibitor resistant melanoma spheroids. Pathol Oncol Res POR. 2020;26(4):2557‐2566. doi:10.1007/s12253-020-00837-9 32613561 PMC7471197

[18] Konieczkowski DJ , Johannessen CM , Abudayyeh O , et al. A Melanoma cell state distinction influences sensitivity to MAPK pathway inhibitors. Cancer Discov. 2014;4(7):816‐827. doi:10.1158/2159-8290.CD-13-0424 24771846 PMC4154497

[19] Misek SA , Appleton KM , Dexheimer TS , et al. Rho‐mediated signaling promotes BRAF inhibitor resistance in de‐differentiated melanoma cells. Oncogene. 2020;39(7):1466‐1483. doi:10.1038/s41388-019-1074-1 31659259 PMC7024013

[20] Cheng C , Yan X , Sun F , Li LM . Inferring activity changes of transcription factors by binding association with sorted expression profiles. BMC Bioinformatics. 2007;8:452. doi:10.1186/1471-2105-8-452 18021409 PMC2194743

[21] Subramanian A , Tamayo P , Mootha VK , et al. Gene set enrichment analysis: a knowledge‐based approach for interpreting genome‐wide expression profiles. Proc Natl Acad Sci U S A. 2005;102(43):15545‐15550. doi:10.1073/pnas.0506580102 16199517 PMC1239896

[22] Yang W , Soares J , Greninger P , et al. Genomics of Drug Sensitivity in Cancer (GDSC): a resource for therapeutic biomarker discovery in cancer cells. Nucleic Acids Res. 2013;41(D1):D955‐D961. doi:10.1093/nar/gks1111 23180760 PMC3531057

[23] Yoshihara K , Shahmoradgoli M , Martínez E , et al. Inferring tumour purity and stromal and immune cell admixture from expression data. Nat Commun. 2013;4:2612. doi:10.1038/ncomms3612 24113773 PMC3826632

[24] Capolongo G , Suzumoto Y , D’Acierno M , Simeoni M , Capasso G , Zacchia M . ERK1,2 signalling pathway along the nephron and its role in acid‐base and electrolytes balance. Int J Mol Sci. 2019;20(17):E4153. doi:10.3390/ijms20174153 31450703 PMC6747339

[25] Dumaz N , Marais R . Integrating signals between cAMP and the RAS/RAF/MEK/ERK signalling pathways. Based on the anniversary prize of the Gesellschaft für Biochemie und Molekularbiologie Lecture delivered on 5 July 2003 at the Special FEBS Meeting in Brussels. FEBS J. 2005;272(14):3491‐3504. doi:10.1111/j.1742-4658.2005.04763.x 16008550

[26] Sensi M , Nicolini G , Petti C , et al. Mutually exclusive NRASQ61R and BRAFV600E mutations at the single‐cell level in the same human melanoma. Oncogene. 2006;25(24):3357‐3364. doi:10.1038/sj.onc.1209379 16462768

[27] Śmiech M , Leszczyński P , Kono H , Wardell C , Taniguchi H . Emerging BRAF mutations in cancer progression and their possible effects on transcriptional networks. Genes. 2020;11(11):E1342. doi:10.3390/genes11111342 33198372 PMC7697059

[28] Yao Z , Yaeger R , Rodrik‐Outmezguine VS , et al. Tumours with class 3 BRAF mutants are sensitive to the inhibition of activated RAS. Nature. 2017;548(7666):234‐238. doi:10.1038/nature23291 28783719 PMC5648058

[29] Wan PTC , Garnett MJ , Roe SM , et al. Mechanism of activation of the RAF‐ERK signaling pathway by oncogenic mutations of B‐RAF. Cell. 2004;116(6):855‐867. doi:10.1016/s0092-8674(04)00215-6 15035987

[30] Murphy BM , Weiss TJ , Holderbaum AM , et al. Functional differences drive the selection of NRAS mutants in melanoma. Cancer Biology. 2021. doi:10.1101/2021.01.15.426808

[31] Summers MG , Smith CG , Maughan TS , Kaplan R , Escott‐Price V , Cheadle JP . *BRAF* and *NRAS* locus‐specific variants have different outcomes on survival to colorectal cancer. Clin Cancer Res. 2017;23(11):2742‐2749. doi:10.1158/1078-0432.CCR-16-1541 27815357

[32] Daud A , Bastian BC . Beyond BRAF in melanoma. Curr Top Microbiol Immunol. 2012;355:99‐117. doi:10.1007/82_2011_163 21826607

[33] Wee P , Wang Z . Epidermal growth factor receptor cell proliferation signaling pathways. Cancers. 2017;9(5):E52. doi:10.3390/cancers9050052 28513565 PMC5447962

[34] Xu Z , Lee CC , Ramesh A , et al. BRAF V600E mutation and BRAF kinase inhibitors in conjunction with stereotactic radiosurgery for intracranial melanoma metastases. J Neurosurg. 2017;126(3):726‐734. doi:10.3171/2016.2.JNS1633 27203149

[35] King AJ , Patrick DR , Batorsky RS , et al. Demonstration of a genetic therapeutic index for tumors expressing oncogenic *BRAF* by the kinase inhibitor SB‐590885. Cancer Res. 2006;66(23):11100‐11105. doi:10.1158/0008-5472.CAN-06-2554 17145850

[36] Tsai J , Lee JT , Wang W , et al. Discovery of a selective inhibitor of oncogenic B‐Raf kinase with potent antimelanoma activity. Proc Natl Acad Sci. 2008;105(8):3041‐3046. doi:10.1073/pnas.0711741105 18287029 PMC2268581

[37] Cox AD , Der CJ , Philips MR . Targeting RAS membrane association: back to the future for anti‐RAS drug discovery? Clin Cancer Res. 2015;21(8):1819‐1827. doi:10.1158/1078-0432.CCR-14-3214 25878363 PMC4400837

[38] Lake D , Corrêa SAL , Müller J . Negative feedback regulation of the ERK1/2 MAPK pathway. Cell Mol Life Sci. 2016;73(23):4397‐4413. doi:10.1007/s00018-016-2297-8 27342992 PMC5075022

[39] Mini E , Nobili S , Caciagli B , Landini I , Mazzei T . Cellular pharmacology of gemcitabine. Ann Oncol. 2006;17(Suppl 5):v7‐12. doi:10.1093/annonc/mdj941 16807468

[40] Fryer RA , Barlett B , Galustian C , Dalgleish AG . Mechanisms underlying gemcitabine resistance in pancreatic cancer and sensitisation by the iMiD^TM^ lenalidomide. Anticancer Res. 2011;31(11):3747‐3756. 22110196

[41] Steinbach A , Clark SM , Clemmons AB . Bosutinib: a novel src/abl kinase inhibitor for chronic myelogenous leukemia. J Adv Pract Oncol. 2013;4(6):451‐455. doi:10.6004/jadpro.2013.4.6.8 25032026 PMC4093452

[42] Parsons SJ , Parsons JT . Src family kinases, key regulators of signal transduction. Oncogene. 2004;23(48):7906‐7909. doi:10.1038/sj.onc.1208160 15489908

[43] Ren D , Hua Y , Yu B , et al. Predictive biomarkers and mechanisms underlying resistance to PD1/PD‐L1 blockade cancer immunotherapy. Mol Cancer. 2020;19(1):19. doi:10.1186/s12943-020-1144-6 32000802 PMC6993488

