# Supplementary material for: A B‐Raf V600E gene signature for melanoma predicts prognosis and reveals sensitivity to targeted therapies
Source: Cancer Med. 2022 Jan 19;11(4):1232–43. doi: 10.1002/cam4.4491 (PMC8855909; doi:10.1002/cam4.4491)
Supplement: Supplementary file 1 — Fig S1‐S4 [file CAM4-11-1232-s002.docx]

**A B-Raf V600E gene signature for melanoma predicts prognosis and reveals sensitivity to targeted therapies**

**Supplementary Figures**

Running head: B-Raf signature for targeted therapies

Kevin Yao^1, ϯ^, Emily Zhou^2, ϯ^, Chao Cheng^3,4,5^*

1. Department of Electrical and Computer Engineering, Texas A&M University, College Station, TX, USA.

2. Department of Biosciences, Rice University, Houston, Texas, USA

3. Department of Medicine, Baylor College of Medicine, Houston, TX 77030, USA

4. Dan L Duncan Comprehensive Cancer Center, Baylor College of Medicine, Houston, TX 77030, USA

5. Institute for Clinical and Transcriptional Research, Baylor College of Medicine, Houston, TX 77030, USA

^ϯ^These authors contributed equally to this work

*Corresponding author

Email: chao.cheng@bcm.edu

Phone: 713-798-3332


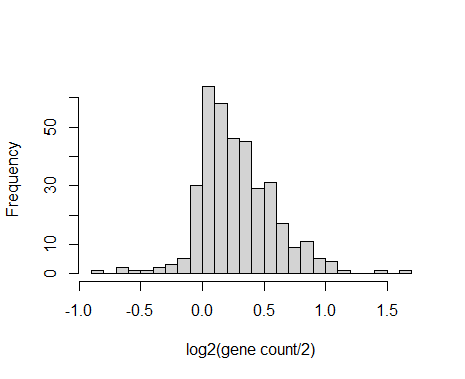


Supplementary Figure 1. Histogram showing the distribution of the copy number variation of BRAF.


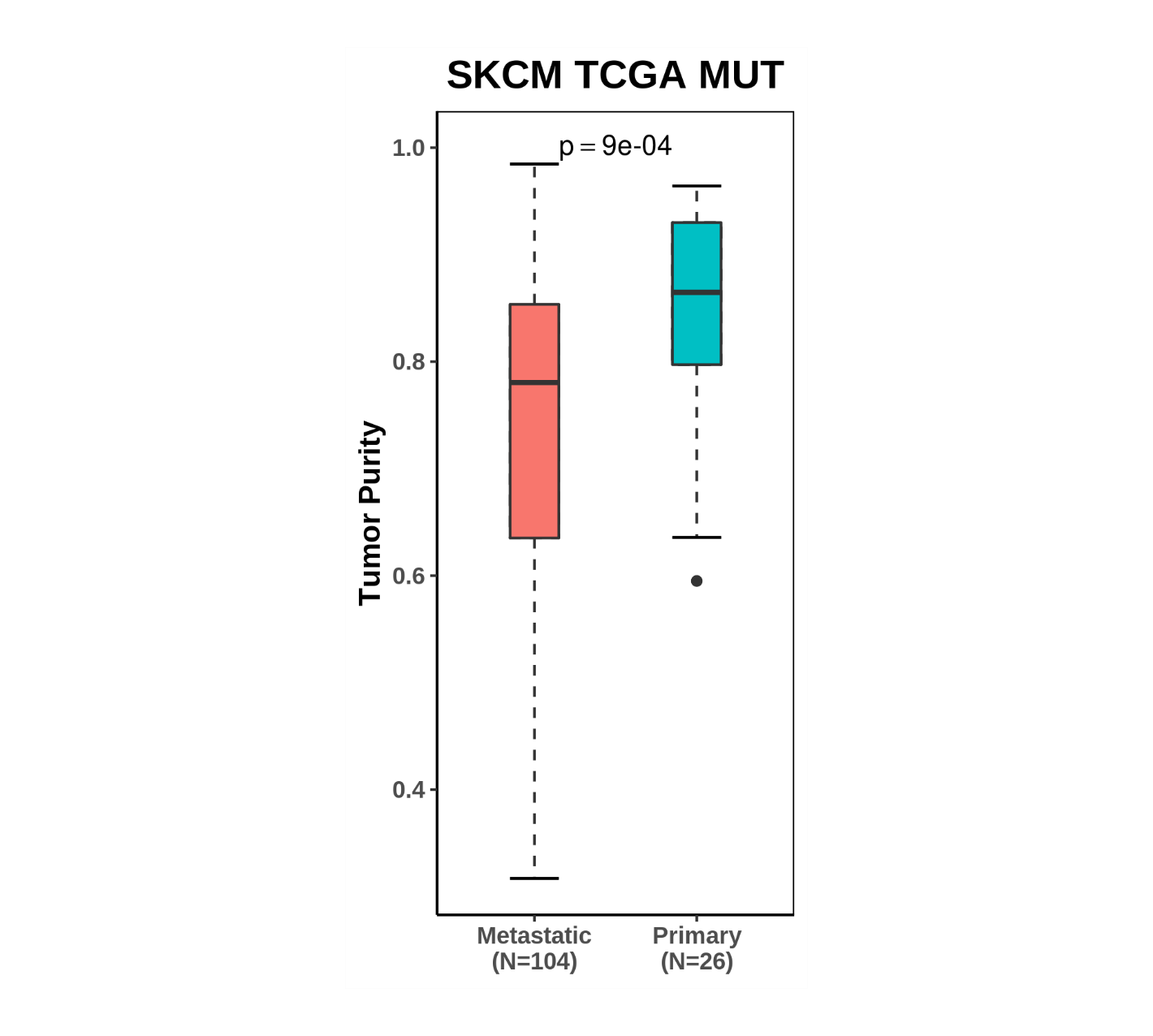


Supplementary Figure 2. TCGA SKCM metastatic samples have lower tumor purity than primary samples due to their more complicated anatomic cell composition. This demonstrates a potential explanation for why primary tumors have higher BRAF scores than metastatic samples in Fig. 1A.


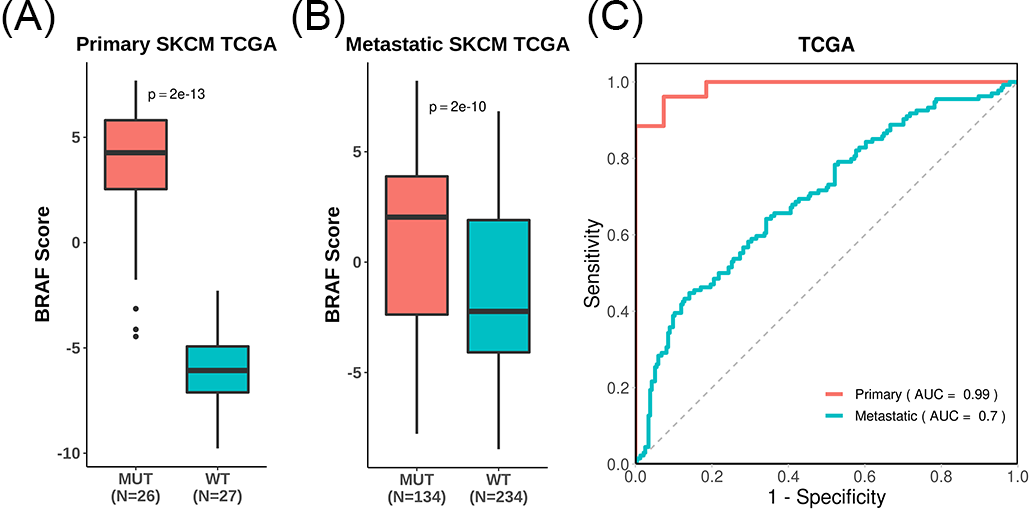


Supplementary Figure 3. The BRAF score was redefined based on other hotspot BRAF mutations reported in COSMIC. (a-b) The score is still significantly higher in V600E mutated samples in both primary and metastatic SKCM samples in the TCGA dataset. (c) BRAF score is still predictive of V600E mutation status.


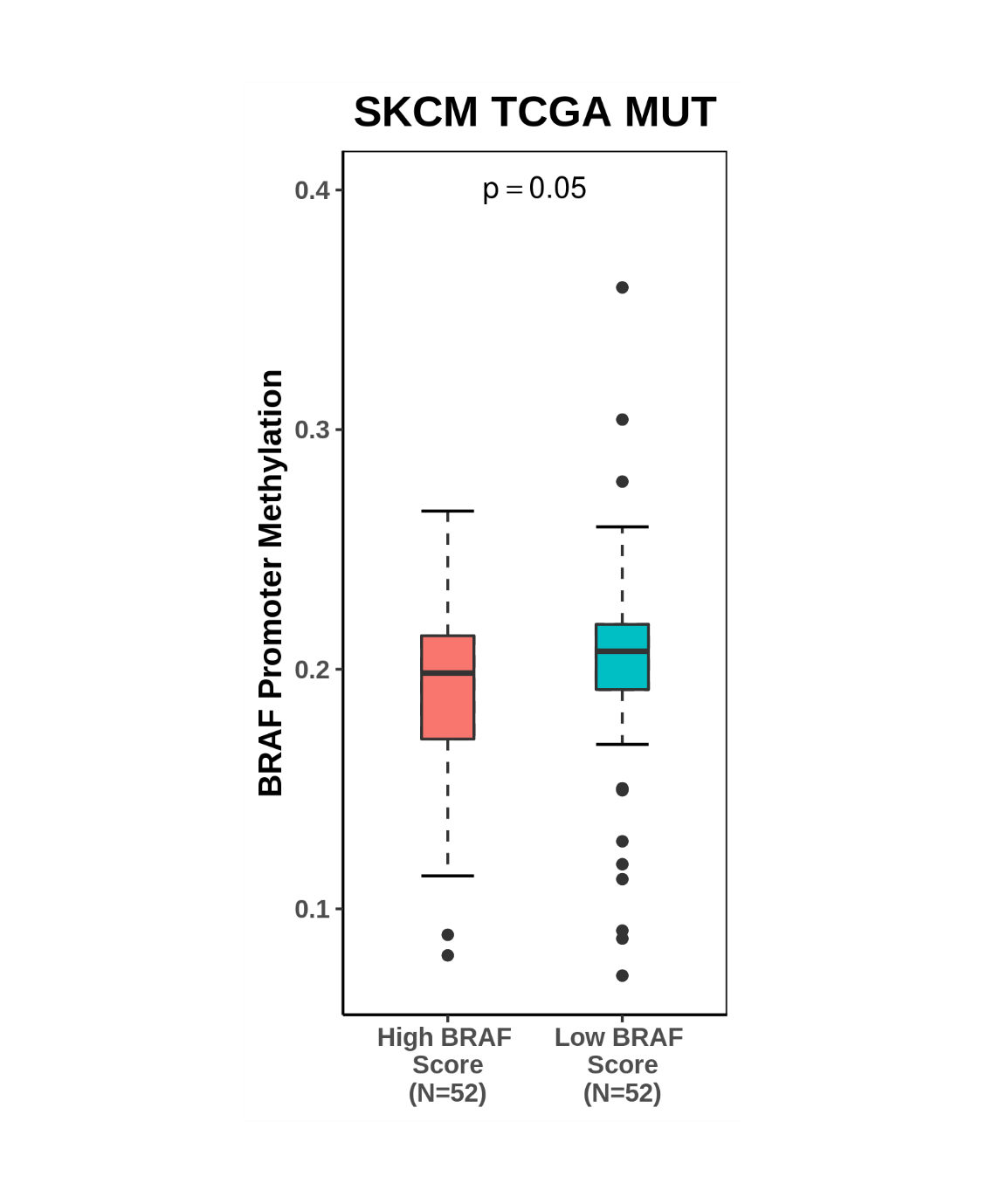


Supplementary Figure 4. Patients in the TCGA SKCM dataset with the V600E mutation and lower BRAF scores also have higher BRAF promoter methylation than those with higher BRAF scores. This could explain why those patients have lower scores to begin with.
